# Investigating the common set of acoustic parameters in sexual orientation groups: A voice averaging approach

**DOI:** 10.1371/journal.pone.0208686

**Published:** 2018-12-10

**Authors:** Sven Kachel, André Radtke, Verena G. Skuk, Romi Zäske, Adrian P. Simpson, Melanie C. Steffens

**Affiliations:** 1 DFG Research Unit Person Perception, Institute of Psychology, Friedrich Schiller University of Jena, Jena, Germany; 2 Department of Social, Environmental, and Economic Psychology, University of Koblenz-Landau, Landau, Germany; 3 Department for General Psychology and Cognitive Neuroscience, Institute of Psychology, Friedrich Schiller University of Jena, Jena, Germany; 4 Department of Otorhinolaryngology, Jena University Hospital, Jena, Germany; 5 Department of German Linguistics, Friedrich Schiller University Jena, Jena, Germany; University of Hull, UNITED KINGDOM

## Abstract

While the perception of sexual orientation in voices often relies on stereotypes, it is unclear whether speech stereotypes and accurate perceptions of sexual orientation are each based on acoustic cues common to speakers of a given group. We ask if the stereotypical belief, that members of the same sexual orientation group share similar acoustic patterns, is accurate to some degree. To address this issue, we are the first to use a novel voice morphing technique to create voice averages from voices that represent extremes of a given sexual orientation group either in terms of actual or perceived sexual orientation. Importantly, averaging preserves only those acoustic cues shared by the original speakers. 144 German listeners judged the sexual orientation of twelve natural-sounding sentence stimuli, each representing an average of five original utterances. Half of the averages were based on targets’ self-ratings of sexual orientation: On a 7-point Kinsey-like scale, we selected targets who were most typical for a certain sexual orientation group according to their self-identifications. The other half were based on extreme ratings by others (i.e., on speech-related sexual-orientation stereotypes). Listeners judged sexual orientation from the voice averages with above-chance accuracy suggesting 1) that the perception of actual and stereotypical sexual orientation, respectively, are based on acoustic cues shared by speakers of the same group, and 2) that the stereotypical belief that members of the same sexual orientation group share similar acoustic patterns is accurate to some degree. Mean fundamental frequency and other common acoustic parameters showed systematic variation depending on speaker gender and sexual orientation. Effects of sexual orientation were more pronounced for stereotypical voice averages than for those based on speakers’ self-ratings, suggesting that sexual-orientation stereotypes exaggerate even those differences present in the most salient groups of speakers. Implications of our findings for stereotyping and discrimination are discussed.

## Introduction

Do they actually all speak the same way? Stereotypes are based on the idea of homogeneity within (sexual orientation) groups [1, 2]. By implication, people belonging to the same sexual orientation group are believed to speak similarly. Contrary to this idea, Zwicky [3] suggested more individuated speaking styles: While one gay man might minimize nasality to stereotypically signal his sexual orientation, another might use a lisp, and a third might raise his voice pitch. Similarly, while one listener might consider voice pitch a distinguishing characteristic between lesbians and straight women, another listener may choose an individual speaker’s pitch variability to determine sexual orientation. In other words, when considering how sexual orientation is expressed in the voice, sociophonetic research on sexual orientation is guided by two opposite implicit assumptions: Either there is a common set of acoustic correlates of sexual orientation [4, 5], or there are multiple different ways of expressing sexual orientation in voices [6, 7]. The present research was designed to test whether there is a common set of acoustic correlates in female and male German speakers besides individual ways of expressing one’s sexual orientation by using an innovative voice morphing technique.

Speech stereotypes are defined as associations between certain social groups (e.g., straight men) and specific speech characteristics (e.g., low-pitched voice; [8–10]). Studies investigating explicit speech stereotypes for male sexual orientation indicate that some stereotypes extend across several cultures, whereas others are culturally specific. For instance, the belief that straight men speak with lower voices than gay men is socially shared across different countries and languages ranging from American English [11] via Puerto Rican Spanish [12] to German [6]. In contrast, the belief that gay men use nasalized speech compared to straight men is as important as the voice pitch stereotype for describing the speech of sexually divergent men in Germany [6], but is expressed less frequently [12, 13], if existing at all in other languages [14, 11]. Regarding female sexual orientation, empirical investigations on explicit speech stereotypes are scarce [15, 16]: interviewees stereotypically characterize lesbians as having a lower-pitched voice than straight women [14]. Because of this gender-based difference in speech stereotypes, more pronounced effects on listeners’ sexual orientation ratings can be expected for gay and straight men than for lesbians and straight women [14].

When determining the accuracy of speech stereotypes in the context of sexual orientation, Judd and Park [17] distinguish at least two different kinds of stereotype accuracy: Stereotypic and dispersion accuracy. Stereotypic accuracy refers to the stereotype content and reflects the correspondence between believed and actual group characteristics (e.g., whether the belief that straight men use lower-pitched voices than gay men is true or not). For the ease of understanding, we henceforth refer to this kind of accuracy as content accuracy. As reviews comparing explicit speech stereotypes and actual speech differences between gay and straight men indicate, content accuracy is low [6]. Although findings on acoustic differences between lesbian/gay and straight speakers are inconclusive, there is a distinct pattern of evidence for the acoustic parameters investigated up to now (see [6] for a recent review): The majority of sociophonetic studies on sexual orientation did not provide acoustic evidence in line with explicit speech stereotypes. For example, while two studies showed straight men to use lower voice pitches than gay men [18, 19], the majority of studies did not find significant group differences [15, 20, 21, 4, 22–25].

Dispersion accuracy is the second kind of stereotype accuracy and refers to the within-group homogeneity belief. It mirrors the correspondence of believed and actual intra-group variability (e.g., whether the belief that most straight men show a certain set of acoustic parameters in contrast to most gay men is true or not). Because stereotypes are generalized representations of social group characteristics, they are assumed to be valid for most members of a given group [17]. Consequently, dispersion accuracy implies that members of one group should produce comparable acoustic characteristics. In contrast to content accuracy, there are only a few sociophonetic studies on sexual orientation dealing with dispersion accuracy [6, 19, 26, 7]). The present study is primarily concerned with dispersion rather than content accuracy.

Sexual-orientation based stereotypes are rooted in gender-inversion beliefs [27]. Accordingly, lesbians and gay men are believed to be less gender conforming than straight women and men. For instance, lesbians compared to straight women are assumed to be more similar to straight men (e.g., more masculine and less feminine than straight women). Hence, lesbians would be expected to show lower values associate with voice pitch (mean f0, lower f0 *SD*, upper and lower f0 boundary) and lower values associated with the acoustic vowel space (mean F1, mean F2, vowel space dispersion) than straight women, based on evidence for acoustic gender differences [28]; vice versa for men. Sociophonetic research on sexual orientation empirically investigated people’s gender inversion theories and tested speech stereotypes. Earlier sociophonetic studies implicitly assumed gay and straight men to be homogeneous groups and hence, assumed a common set of acoustic correlates of sexual orientation given a certain linguistic and cultural background (e.g., [4, 5]). This is indicated by the simple comparison of mean acoustic parameters between sexual orientation groups while disregarding any fine-grained sexual orientation measurement that might have revealed intra-group variability. This approach has been pursued up to the present day, although most studies do suggest that within-group differences exist. For instance, in spite of above-chance accuracy, not every man was correctly judged as gay or straight based on voice recordings [22, 29]. Furthermore, attempts for explaining acoustic differences within groups have found their way into sociophonetic research on sexual orientation (e.g., [30]). Crucially, evidence for intra-group variability has been found [6, 7], which hints at multiple different ways of acoustically coding actual sexual orientation and hence, suggests dispersion inaccuracy as one aspect of stereotype inaccuracy.

Taken together, previous research on acoustic cues to sexual orientation in voices reflects two implicit assumptions that refer to dispersion (in)accuracy: Either there is a common set of acoustic correlates of sexual orientation given a certain linguistic and cultural background, or there is a variety of ways of coding sexual orientation in voices. Based on the first assumption speakers belonging to one sexual orientation group are considered as rather homogeneous and systematically different from other sexual orientation groups with respect to specific acoustic parameters (e.g. [4, 5]). Studies following this approach assume, for instance, that straight women use, on average, a higher fundamental frequency and a more expanded vowel space compared to lesbians and bisexual women. According to this perspective, there should be a uniform set of acoustic parameters for each sexual orientation group in a certain language and culture, leading to pronounced differences between sexual orientation groups. Listeners should be able to perceive these acoustic differences. This is in line with previous voice-based evidence showing that straight people were rated as straighter compared to lesbians/gay men [31, 15, 32, 21]. In contrast, the second assumption emphasizing intra-group diversity considers speakers belonging to one sexual orientation group (e.g., straight men) to use different acoustic cues to code their sexual orientation (see [3, 20, 33, 34]). Studies following this approach implicitly assume [6, 7], for instance, that one man indicates his straightness by using a low fundamental frequency while another applies a restricted vowel space in a specific situational context. According to this perspective, strictly speaking, on average there should be no acoustic differences between sexual orientation groups. To the best of our knowledge, no voice-based perception study has investigated bisexual women and men as a category separate from lesbian/gay speakers. Hence, in the present research we included three sexual orientation groups: lesbian/gay, bisexual, and straight speakers.

Previous sociophonetic studies on sexual orientation have routinely aggregated individual acoustic parameters within groups and compared them between groups, although even early studies recommended choosing methods beyond mean comparisons [22]. Applying simple mean comparisons as a tool for contrasting acoustic parameters of different sexual orientation groups allows for the investigation of content (in)accuracy. However, by using this method, no conclusion can be drawn about dispersion (in)accuracy and hence, whether a common acoustic set exists for speakers belonging to the same sexual orientation group. Moreover, this conventional approach does not answer the question whether common voice characteristics drive the perception of sexual orientation because different acoustic features could have influenced the perception of different speakers.

To test for dispersion (in)accuracy, in the present research we created naturally sounding voice averages across several speakers uttering the same neutral German sentence using a novel n-way morphing approach [35]. While voice morphing has not been used in research on sexual orientation, this powerful technique has been employed to study the perception of vocal gender and age [36–38], emotional prosody [39, 40], identity [41] or attractiveness [42, 43]. Since voice morphing requires ample phonetic expertise and is technically challenging, most researchers have used simple vowel-consonant-vowel stimuli (e.g., <igi>), rather than more complex sentence utterances, and have usually averaged voices of no more than two speakers at a time. We created, for the first time, high-quality voice averages across several speakers uttering a whole sentence. We thereby minimized possible individual differences between speakers of the same sexual orientation group and simultaneously highlighted the acoustic similarities within each sexual orientation group. Similar to statistical averaging, a voice average can be regarded as the best vocal representation of a set of individual voices. Hence, averaging results in one naturalistic sounding voice which is characterized by those acoustic features that are common to members of a specific sexual orientation group, because any speech characteristics produced by only one speaker would be averaged out or reduced in saliency (see above). Note that the morphing procedure would therefore not preserve putative person-specific markers of sexual orientation. Importantly, the voice averages can be presented to and judged by listeners to assess the acoustic and perceptual correlates of sexual orientation.

The assumption that listeners who judge sexual orientation use a consistent set of acoustic cues common among speakers of a given sexual orientation would be supported if sexual orientation information could still be perceived in voice averages. This would suggest that the stereotypical belief that members of one group are homogeneous regarding their (speech) characteristics [1, 2] is accurate to some degree and therefore, indicates dispersion accuracy. Moreover, this set of cues could represent valid signals to sexual orientation and/or speech stereotypes held by listeners.

According to one of the oldest hypotheses of stereotypes, stereotypes contain a “kernel of truth” (see [44]), but exaggerate differences between social groups [45]. We tested this hypothesis by contrasting two different sets of speakers. First, we used single voices of speakers who identified their sexual orientation themselves (i.e., self-rated sexual orientation) and second, we selected recordings of speakers who were perceived as very typical for one sexual orientation group in previous studies (i.e., sexual orientation rated by others; [46, 47]). Voices that were previously rated by others as very typical contain stereotypical information because listeners align the acoustic information they hear with their concepts of what a person with a certain sexual orientation sounds like (see [48], for research on stereotype activation in the visual domain). Overall, we expected rather small effects due to recent findings supporting the assumption that there are multiple different ways of vocally coding sexual orientation [6, 7]. That is why we included the stereotypical voices, because we assumed them to show more pronounced effects compared to voices of speakers who rated their sexual orientation themselves. As we found acoustic correlates of actual and perceived sexual orientation to be different [6], we assumed voice averages of self- and other-rated sexual orientation to rely on different sets of acoustic cues. Moreover, we wanted to give some hints to possible acoustic parameters that may provoke listeners’ sexual orientation judgments of the voice averages. Hence, we analyzed acoustical parameters commonly investigated in sociophonetic studies on gender [28] and sexual orientation [49] in order to determine which of them differ between sexual orientation groups. These include mean f0 [18, 19, 50, 26], vowel space expansion, vowel space shift, and F1 and F2 values in single vowels [30, 15, 24, 25]. When analyzing intonational features and vowel space characteristics, we investigate whether there are descriptive group differences in mean acoustic parameters and hence, focus on content accuracy in addition to dispersion (in)accuracy.

## The present study

The aim of the present rating study was to test whether the same voice characteristics drive the perception of sexual orientation of different speakers. In order to increase the probability of finding common acoustic features, if these exist at all, we created voice averages using an innovative n-way morphing approach [35]. We created twelve natural-sounding sentence stimuli, each representing an average of five original utterances. The voice averages are therefore represent of maximally different groups and, at the same time, typical of the respective groups. They were maximally different from each other in that speakers belonging to divergent sexual orientation groups showed very distinct sexual orientation ratings, whereas speakers belonging to the same sexual orientation group showed fairly homogeneous sexual orientation ratings. Maximizing sexual orientation differences between speakers belonging to different sexual orientation groups and minimizing differences within each sexual orientation group resulted in typical voice averages. Half of the averages were based on targets’ self-ratings of sexual orientation (as 1 = lesbian/gay, 4 = bisexual women/men, or 7 = straight women/men). The other half were based on most extreme ratings by others (i.e., on speech-related sexual-orientation stereotypes).

Based on the assumption that different speakers use a common set of acoustic cues to signal sexual orientation, and that listeners can use that set of cues, we tested the following hypotheses:

1. Voice averages of straight speakers are judged as straighter than voice averages of bisexual speakers, who are in turn judged as straighter than voice averages of lesbian/gay speakers.
2. These differences are found for both female and male speakers and more pronounced for male speakers because of stronger stereotypes regarding male than female sexual orientation.
3. In line with the “kernel of truth” hypothesis of stereotyping, effects should be more pronounced for sexual orientation groups based on ratings by others (i.e, for stereotypical voice averages) than for sexual orientation groups based on self-ratings.

## Method

### Ethics statement

The experiment was conducted in accordance with the Declaration of Helsinki and was approved by the Ethics Committee of the University of Jena, Faculty of Social and Behavioral Sciences (approval number FSV 12/02). Participants were initially informed that participation was anonymous, voluntary, and could be canceled anytime during the experiment without fearing any negative consequences. If they still agreed to participate, they clicked a respective button that initiated the study.

### Speakers

Initially, 111 (57 female, 54 male, *M*_*age*_ = 24.08, *SD*_*age*_ = 2.55, age range 19–30 years) speakers were recorded under standardized conditions in a sound-treated room [6, 7]. From a total of twenty different sentences, the target sentence for this study was selected: *Der Tag ist sehr lang geworden*. (/de:rta:kɪstze:rlaŋɡəvᴐrdən/; “It has been a very long day.”). This sentence was chosen because it contains examples of features that have commonly been found to differ based on gender and sexual orientation, for example, vowel space dimensions in the range of front-back, close-open vowels; sibilant measures for /s z/; VOT in the initial fortis plosive of *Tag*. The sentence was also considered to be suitable as a basis for creating averages of several utterances: it is segmentally relatively simple and one would not expect much inter-individual variation in the segmental realization. To be able to choose the best realization (e.g. no breathing, no clicking), each sentence was spoken three times by the speaker in a neutral way.

All speakers provided information on their sexual orientation by using two measures. First, the speakers were asked to self-identify their sexual orientation on a 7-point *Kinsey-like scale* (1, exclusively lesbian/gay; 4, bisexual, and 7, exclusively straight; [51]). Second, the speakers filled out a more objectifiable instrument that measures sexual orientation towards women and men separately by using four items each (physical attraction, sexual fantasies, romantic emotions, and sexual interaction; for details see [52]). For example, speakers were asked to index their sexual attraction to women. Items were rated on a 7-point scale in order to answer the question “How often have you experienced …” (“1 = never”, “7 = always”). For each speaker, we computed the mean for sexual orientation towards men and women separately and calculated the mean difference for the two scales that served as a S*exual Orientation Index*. Positive scores indicate a stronger sexual orientation towards men and negative scores a stronger sexual orientation towards women.

### Selection of speakers for the present study

To create voice averages, single voice recordings were selected according to four criteria.

**Fig 1:**
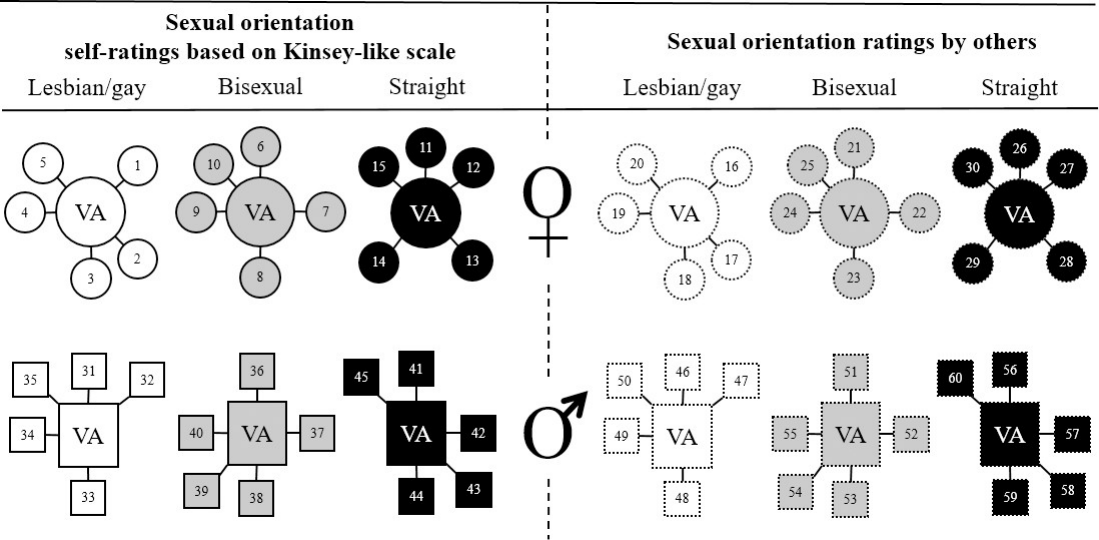
Voice averages (VA) originated from individual voices (1–60) of 5 female and male speakers who were lesbian/gay, bisexual, and straight based on self-ratings or ratings by others.

- Recordings of five speakers were selected to create one voice average (e.g., five voices of women who were rated by others as bisexual) because we first expected five speakers to be sufficient in order to minimize individual speech characteristics, and second, the preparation of a total number of 60 utterances for morphing is time-consuming and challenging. Including more speakers would be in opposition to the aim of maximizing differences between voice averages (e.g., no more speakers were available who rated themselves as bisexual).
- We selected speakers with the most typical sexual orientation scores regarding self-ratings or ratings by others in order to maximize acoustic information indicating sexual orientation (e.g., extreme ratings for lesbian/gay and straight speakers and intermediate ratings for bisexual speakers).
- Due to different numbers of speakers possibly available for each voice average (e.g., few men rated themselves as bisexual but a lot were rated as bisexual by others), we first selected voices for averages where small numbers of speakers (vs. high numbers) were available.
- In order to avoid redundant signals and to maximize contrasts between voices, a given voice was not selected for more than one voice average (see Fig 1).

As illustrated in Fig 1, two sets of speakers were selected for the purpose of the current study. A first set of 30 speakers was selected based on the speakers’ self-ratings (15 female, 15 male; 5 in each of the groups: lesbian/gay, bisexual, or straight, respectively). The primary differentiation was done according to the Kinsey-like scale because it provides a clearly identity-based assignment as either exclusively lesbian/gay or straight, or very close to the scale’s center (i.e., bisexual). As a secondary differentiation, we used the more fine-grained *Sexual Orientation Index*. Whenever more than five speakers had the same Kinsey-like score, those were chosen whose Sexual Orientation Index was most typical for a given sexual orientation group. In this way, most extreme groups of speakers were created that differed maximally regarding self-rated sexual orientation.

A second set of 30 speakers was selected based on sexual orientation rated by others (15 female, 15 male; five in each of the groups: lesbian/gay, bisexual, or straight, respectively). From the whole pool of 111 speakers, 18 lesbians, gay men (Kinsey-like scores 1–2), straight women and men (Kinsey-like scores: 6–7) each had been rated as either lesbian/gay or straight based on sentence recordings by two independent samples of a total of 216 participants (121 women, 95 men, *M*_*age*_ = 28.12, *SD*_*age*_ = 10.53, age range 18–71 years), in two different studies ([46] and an unpublished Master’s thesis [47]). We consider these stimuli as stereotypical. Based on these ratings, we computed mean ranks and mean rank differences between the two independent rater samples. Selection for the “ratings by others” condition was primarily based on mean ranks. Whenever two speakers showed the same mean ranks, those with smaller mean rank differences were preferred because raters’ perceptions regarding that speaker were more similar. In this way, maximally different groups regarding speech stereotypes were created. Self-ratings and ratings by others for each voice average are listed in Table 1.

**Table 1:** Overview of sexual orientation ratings for all voice averages.

	5-voice female averages, based on…						5-voice male averages, based on…					
	…self-ratings			…ratings by others			…self-ratings			…ratings by others		
	L	B	S	L	B	S	G	B	S	G	B	S
Age	23.20	23.00	23.00	23.20	25.00	23.20	25.80	24.60	25.60	24.80	24.20	23.40
*Self-ratings of sexual orientation*												
Kinsey-like scale^a^	1.00	4.00	7.00	2.80	5.20	4.80	1.00	4.40	7.00	2.60	2.60	4.80
Sexual Orientation Index^a^	-5.25	.00	4.40	-2.30	2.25	.85	5.65	-.10	-5.75	3.45	2.45	-1.75
*Ratings of sexual orientation by others*												
Perceivedstraightness^b^	.80		.88	.62	.80	.90	.65	.70	.66	.31	.66	.87
Meanranks^b^	17.50		26.83	3.40	15.20	31.50	14.00	16.5	19.83	3.00	13.75	32.00
Mean rank differences^b^	3.67		-.33	-.40	-1.20	-.60	6.00	13.00	.33	.00	.60	-1.60

Abbreviations: B = bisexual wo/men, G = gay men, L = lesbians, S = straight wo/men, SD = standard deviation, Kinsey-like scale ranged from 1 –“exclusively lesbian/gay” via 4 –“equally lesbian/gay and straight” to 7 –“exclusively straight”. Sexual Orientation Index ranged from -7 –“sexually oriented towards women only” via 0 –“sexually oriented towards women and men” to 7 –“sexually oriented towards men only”. Perceived straightness indicates mean relative numbers of perceptions as straight in both pre-ratings ranging from 0 –“judged as straight by 0%” to 1 –“judged as straight by 100%”. Mean ranks ranged from 3 –“voices were on average located at the lesbian/gay end of the perceived sexual orientation distribution” via 18.50 –“voices were on average located at the bisexual area of the perceived sexual orientation distribution” to 34 –“voices were on average located at the straight end of the perceived sexual orientation distribution”. Mean rank differences close to 0 indicate that voices were rated as very similar on average in both pre-studies. Note that we did not depict standard deviations for self-ratings and ratings by others because of small sample sizes (*n* = 5 for each voice average).

^a^*n* = 5.

^b^*n* for calculating mean ranks and mean rank differences ranged from 0 for women who rated themselves as bisexual to 4 for men who rated themselves as gay, because not every speaker who was selected for voice averaging based on self-ratings was selected for ratings by others.

### Stimuli

Preparation. For each of the selected 60 speakers, we chose the utterance (out of three) judged to have a segmental make-up most appropriate for morphing. Whenever signal quality was comparable across the three repetitions of the sentence, we selected the one that was contained in the pre-ratings (*n* = 37). Whereas this could be considered a methodological shortcoming, we argue that straightness should be invariant for the same speaker of the same utterance in the same situation. Moreover, voice averaging would minimize such subtle intra-group differences. 50 ms of silence was inserted before and after each utterance. Overall, 32 artefacts (e.g., clicks, smacking noises, doubled and coarticulatory plosives) were manually deleted before morphing. Additionally, selected recordings were root-mean-square normalized at 70 dB SPL using Praat [53].

**Fig 2:**
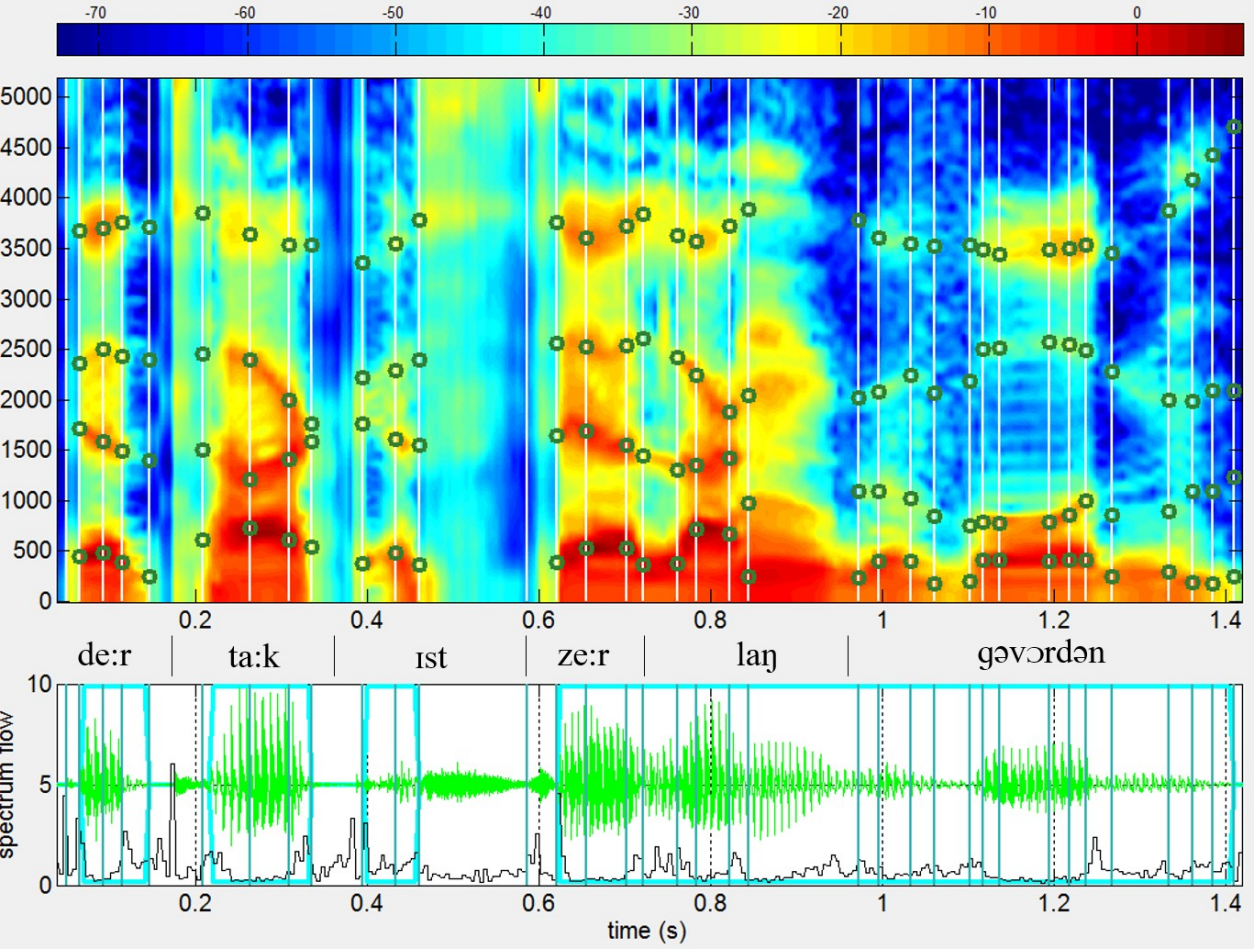
TANDEM STRAIGHT view including time (parallel lines) and frequency anchors (dots on the lines) of the chosen sentence <Der Tag ist sehr lang geworden> uttered by an one speaker.

Voice averaging. Voice averaging was performed using the novel n-way morphing approach of the speech analysis, modification and resynthesis framework TANDEM-STRAIGHT [35]. STRAIGHT-based morphing decomposes a speech signal into three mutually independent parameters, relating to the source (i.e. f0 and aperiodicity), and the filter (the spectral envelope) information. “The interference-free representations of instantaneous frequencies and power spectra with a spectral envelope recovery procedure enable virtually perfect suppression of interferences between parameters which yields in perceptually identical resynthesized speech from only three parameters when no parameter modification is introduced.” [Schweinberger et al. 2013] (see [35] for technical details). To average n voices in a single step, a preprocessing, i.e. a manual mapping of the same time- and frequency-anchors of corresponding key-features in the spectrograms of the original utterances (e.g., onsets and offsets of phonemes, vowel transitions, see Fig 2), has to be done for all utterances to be included in an average. Each of the n voice representations is weighted by a factor of 1/n, before the synthesis output yields into a naturally sounding voice morph. Please refer to S1–S12 Files to listen to all of the twelve voice averages.

A total of twelve 5-voice averages were created for every condition (sexual orientation group: lesbian/gay vs. bisexual vs. straight x speaker gender: female vs. male x rating basis: self- vs. other-ratings). For the present sentence-morphs 36 time-anchors were chosen per voice sample; frequency anchors were placed at center frequencies of formants (F1-F4, where detectable) at each time-anchor location. Two time anchors marked voiceless parts of the utterance and thus did not contain frequency anchors.

### Listeners

The listeners were recruited via a students-only mailing list at the University of Koblenz-Landau (on January 8, 2016). The study was announced as an investigation of the prejudice that speakers’ sexual orientation could be detected based on their voices. Overall, 237 participants took part in the online listening experiment and 155 completed it. Data from 10 listeners were excluded because they were non-native German speakers, reported hearing impairments, or had participated in previous studies on the judgement of sexual orientation based on voices. Moreover, we excluded data from one person whose gender was described as neither male nor female because we wanted to test for possible effects of listener gender. Accordingly, data from 144 listeners (110 female, 34 male, *M*_*age*_ = 23.13, *SD*_*age*_ = 3.67, age range 18–43 years) were analyzed. According to our study announcement, every fifth participant received 10€ recompense.

### Design and procedure

A total of twelve voice averages were available as stimuli: three sexual orientation groups (lesbian/gay vs. bisexual vs. straight), two speaker genders (female vs. male), and two types of ratings (self-rated vs. rated by others). Listeners heard a single test voice (that was not part of any voice average) and were instructed to select a comfortable sound level. During the experiment, the listeners were asked to judge each of the twelve voice averages regarding perceived straightness on a 7-point scale ranging from 1 “very lesbian”/“very gay” via 4 “bisexual” to 7 “very straight” by mouse-click. Listeners were told that the stimuli displayed the whole range of very lesbian/gay to very straight sounding voices. Male and female voice averages were presented in separate blocks, to avoid switching between gender-related standards of voice perception. Listeners were randomly assigned to one of two block orders (i.e., 76 listeners heard female voice averages first, the other 68 heard male voice averages first). Within each block, stimuli were presented to each listener in an individual random order. Mean perceived sexual orientation was computed for each voice average with higher scores indicating higher perceived straightness. Listeners reported sociodemographic and other relevant characteristics at the end of the study.

### Acoustic analysis

For each of the voice averages typical acoustical features were measured using Praat [53]. These included f0 measures (mean, *SD*, 2.5^th^ and 97.5^th^ percentile indicating lower and upper f0 boundary) and mean formant frequencies (F1-F2) of three vowels (corresponding to the inner section, i.e. 25^th^ to 75^th^ percentile: /a:/ in “Tag”, /ɪ/ in “ist”, and /ᴐ/ in “geworden”). F0 was tracked every 10ms for the whole duration of each voice average. In order to present valid indicators for lower and upper f0 boundary that are unaffected by statistical outliers, we used 2.5^th^ and 97.5^th^ percentiles. F1 and F2 were tracked every 6 ms during half of a vowel’s duration centered around the vowel midpoint in order to minimize co-articulatory effects of adjacent consonants. From the F1 and F2 values, we computed vowel space dispersion and vowel space shift. Vowel space dispersion is defined as the mean Euclidian distance of the three vowels from the center of the vowel triangle [54]; vowel space shift is indicated by mean F1 and F2 across the three vowels (see Table 2).

**Table 2:** Overview on acoustic parameters for all voice averages (in Hz).

	5-voice female averages, based on…						5-voice male averages, based on…					
	…self-ratings			…ratings by others			…self-ratings			…ratings by others		
	L	B	S	L	B	S	G	B	S	G	B	S
*Fundamental frequency features*												
f0 mean	209^a^	190	198	176^b^	171	206	122^b^	113	106	117^b^	109	106
f0 *SD*	10^b^	10	19	20^b^	17	21	15^b^	11	10	12^a^	10	17
f0 2.5th percentile	191^a^	175	168	134^b^	146	174	99^b^	95	85	100^b^	93	81
f0 97.5th percentile	235^b^	210	231	203^b^	203	239	144^b^	134	126	137^b^	125	135
*Vowel space characteristics*												
F1 mean	624^b^	612	582	562^b^	600	625	520^a^	559	545	526^b^	545	500
F2 mean	1403^a^	1436	1422	1363^b^	1408	1408	1306^b^	1238	1173	1292^b^	1269	1183
Vowel space dispersion	359^a^	410	387	355^b^	405	360	371^b^	394	368	345^b^	398	328

Abbreviations: B = bisexual wo/men, f0 = fundamental frequency, F1 = first formant frequency, F2 = second formant frequency, G = gay men, L = lesbians, S = straight wo/men, SD = standard deviation.

^a^Acoustic differences between lesbian/gay and straight speakers that were in line with speech stereotypes about sexual orientation groups drawn from lay gender inversion theory.

^b^Acoustic differences between lesbian/gay and straight speakers that were *NOT* in line with speech stereotypes about sexual orientation groups drawn from lay gender inversion theory.

## Results

### Perception of sexual orientation

A 3 x 2 x 2 x 2 x 2 ANOVA was performed on perceived straightness, with three within-subject factors of interest (speaker sexual orientation, speaker gender, and rating basis), and two between-subject control factors (listener gender and block order; cf. Design section). The probability for correctly rejecting the null hypothesis in case there are true differences is 1 – β = .93 given medium effect sizes. As expected, this analysis did not reveal any statistically significant main effects or interactions involving listener gender or block order (all *p*s> .056).

The analysis revealed main effects of speaker sexual orientation, *F*(2, 139) = 112.38, *p*< .001, η_p_^2^ = .62, and rating basis, *F*(1, 140) = 45.65, *p*< .001, η_p_^2^ = .25. Main effects were qualified by three first-order interactions of speaker sexual orientation x rating basis, *F*(2, 139) = 29.34, *p*< .001, η_p_^2^ = .30, speaker sexual orientation x speaker gender, *F*(2, 139) = 8.91, *p*< .001, η_p_^2^ = .11, and speaker gender x rating basis,*F*(1, 140) = 8.64, *p* = .004, η_p_^2^ = .06, all other effects: *F*(2, 140) < 1.91, p ≥ .152, η_p_^2^ ≤ .03. For a detailed analysis of which voice averages differ from each other, we applied simple-effects tests with Bonferroni adjustment.

**Fig 3:**
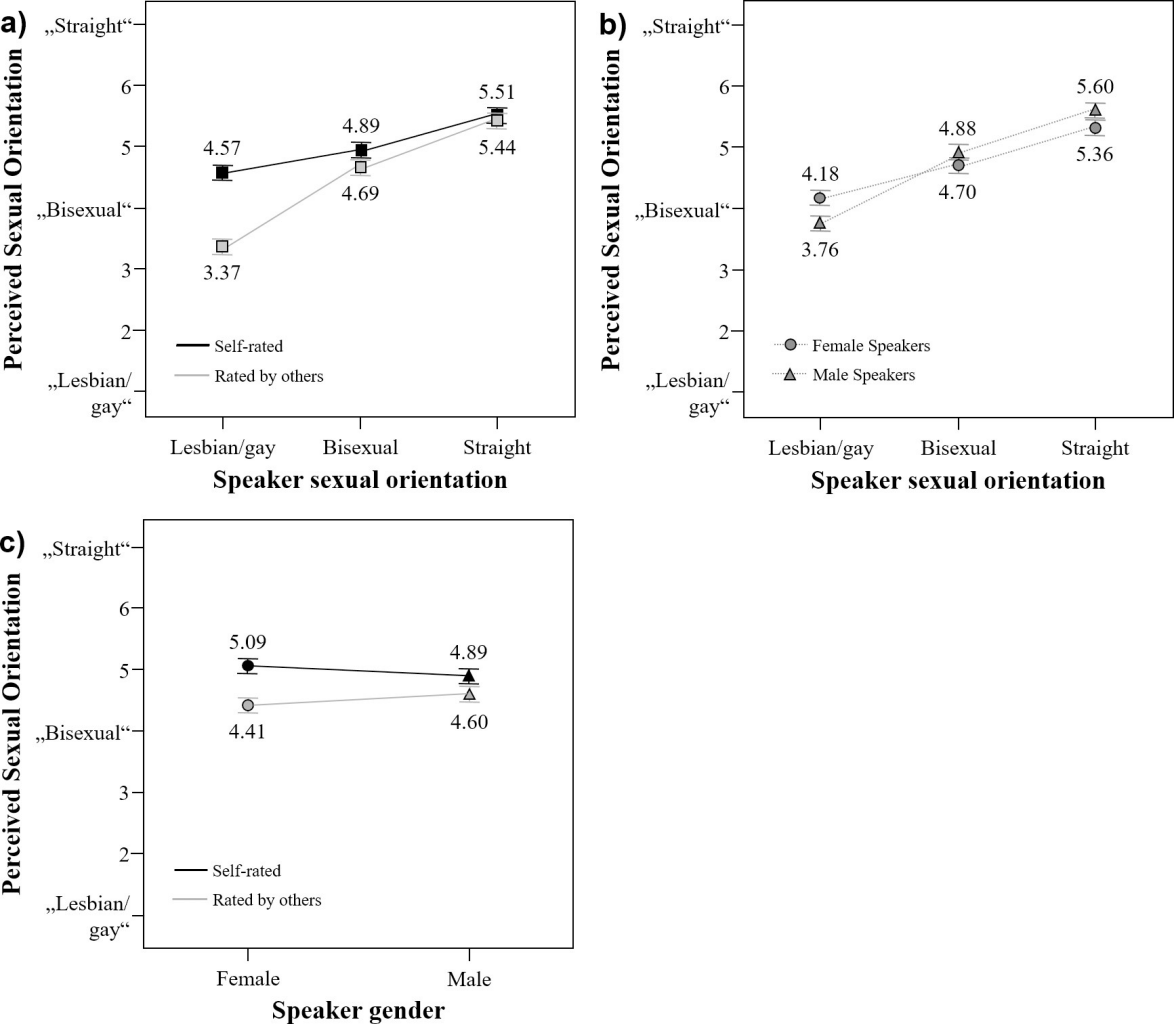
a. First-order interaction effects of speaker sexual orientation x rating basis in perceived sexual orientation of voice averages. b. First-order interaction effects of (b) speaker sexual orientation x speaker gender in perceived sexual orientation of voice averages. c. First-order interaction effects of speaker gender x rating basis in perceived sexual orientation of voice averages.

Fig 3A shows the interaction of speaker sexual orientation x rating basis. Straight voice averages were perceived as straighter compared to bisexual voice averages that were, in turn, perceived as straighter than lesbian/gay voice averages. This was true for ratings by others, *F*(2, 139) = 116.53, *p* < .001, η_p_^2^ = .63 (all pair-wise *p*s ≤ .001), and self-ratings, *F*(2, 139) = 32.33, *p* < .001, η_p_^2^ = .32 (all pair-wise *p*s ≤ .021), supporting Hypothesis 1. According to effect sizes, the differences between sexual orientation groups were much more pronounced for voice averages based on ratings by others (i.e., sexual-orientation stereotypes) than self-ratings (i.e., actual differences between most extreme groups), in line with Hypothesis 3. Moreover, only lesbian/gay voice averages differed regarding the rating basis: Self-rated lesbian/gay voice averages were perceived as straighter than those rated by others, *F*(1, 140) = 89.55, *p* < .001, η_p_^2^ = .39. For bisexual and straight voice averages, rating basis had no effect, *F*s(1, 140) ≤ 2.41, *p*s ≥ .123, η_p_^2^s ≤ .02. This finding partially (i.e., for lesbian/gay voice averages) supports our initial assumption that even after voice averaging, effects of perceived sexual orientation were more pronounced when single voices were previously rated as very typical for one sexual orientation group by others compared to self-ratings. In other words, speech stereotypes of lesbians/gay men exaggerate differences found for self-ratings.

Fig 3B shows the same pattern as Fig 3A but illustrates the interaction of speaker sexual orientation and speaker gender. Straight voice averages were perceived as straighter than bisexual voice averages, which, in turn, were perceived as straighter in contrast to lesbian/gay voice averages; that was true for both male, *F*(2, 139) = 85.48, *p* < .001, η_p_^2^ = .55 (all pair-wise *p*s< .001) and female speakers, *F*(2, 139) = 44.61, *p* < .001, η_p_^2^ = .39 (all pair-wise *p*s < .001), in line with Hypotheses 1 and 2. According to effect sizes, differences in perceived sexual orientation were more pronounced for male than female sexual orientation groups. Furthermore, gay voice averages were perceived as less straight compared to lesbian voice averages, *F*(1, 140) = 8.70, *p* = .004, η_p_^2^ = .06. No gender difference occurred for bisexual voice averages, *F*(1, 140) = 1.74, *p* = .190, η_p_^2^ = .01, and straight men’s voice averages were perceived as straighter compared to straight women’s voice averages, *F*(1, 140) = 4.13, *p* = .044, η_p_^2^ = .03. Hence, fully supporting Hypothesis 2, differences were more pronounced for male than female speakers.

Fig 3C illustrates the interaction of speaker gender and rating basis. Voice averages based on self-ratings were perceived as straighter compared to voice averages based on ratings by others; this was true for female, *F*(1, 140) = 40.09, *p* < .001, η_p_^2^ = .22, and male voice averages, *F*(1, 140) = 11.53, *p* = .001, η_p_^2^ = .08, but differences were more pronounced for female than male voice averages according to effect sizes. Within female speakers, *F*(1, 140) = 2.75, *p* = .100, η_p_^2^ = .02, and male speakers, *F*(1, 140) = 3.54, *p* = .062, η_p_^2^ = .03, voice averages based on self-ratings vs. ratings by others did not differ significantly.

Summing up, sexual orientation information was still perceived after voice averaging of groups maximally different in their sexual orientation: Straight voice averages were perceived as straighter compared to bisexual voice averages that were still perceived as straighter compared to lesbian/gay voice averages. The order was unaffected by rating basis and speaker gender. However, the differences in perceived sexual orientation between sexual orientation groups were more pronounced in ratings by others (vs. self-ratings) and in male speakers (vs. female speakers). Thus, the most typical members of one sexual orientation group seem to share a consistent set of acoustic cues that listeners use to judge their sexual orientation, but speech stereotypes exaggerate existing group differences.

### Acoustic correlates of sexual orientation

Having demonstrated that a consistent set of acoustic cues drives the perception of extreme groups’ sexual orientation, we asked which acoustic characteristics differ between sexual orientation groups and may thus lead to differences in perceived straightness. Acoustic values for fundamental frequency features (mean f0, f0 *SD*, 2.5^th^ percentile, 97.5^th^ percentile) and vowel space characteristics (mean F1, mean F2, vowel space expansion) for every voice average can be seen in Table 2. On a descriptive level, 22 out of 28 possible acoustic differences were in line with speech stereotypes drawn from lay gender inversion theory when excluding bisexual voice averages: Lesbian voice averages showed lower acoustic values than straight women’s voice averages by trend, while voice averages of gay men displayed higher acoustic values than straight men’s voice averages (see Table 2). The number of stereotype-congruent differences was twice as high for women’s voice averages based on ratings by others (*n* = 7) compared to voice averages based on self-ratings (*n* = 3), whereas rating basis did not affect the number of differences for men’s voice averages (*n* = 6 both for self-ratings and ratings by others).

## Discussion

The present research tests the (in)accuracy of speech stereotypes in the context of sexual orientation. Compared to previous research, the present study is particularly concerned with dispersion (in)accuracy (e.g., Do speakers of the same sexual orientation group use similar acoustic patterns as stereotypically expected?) instead of content accuracy (e.g., Do straight men have a lower-pitched voice than gay men?). Hence, we ask whether speakers belonging to the same sexual orientation group exhibit a common acoustic set besides using individual ways of vocally projecting their sexual orientation. We applied acoustic morphing to minimize possible inter-individual acoustic differences between voices of speakers belonging to the same group and to strengthen possible acoustic similarities between them. We created separate voice averages of speakers who either identified themselves strongly with a certain sexual orientation, or who most listeners perceived as having a certain sexual orientation. Overall, voice averages of straight speakers were perceived as straighter than those of bisexual speakers who, in turn, were perceived as straighter compared to lesbian/gay speakers, which was true for female and male, as well as for self- and other-rated voice averages. Hence, after minimizing inter-individual acoustic differences within sexual orientation groups, sexual orientation information can still be perceived in voice averages. Whereas differences in perceived sexual orientation were more pronounced for male than female sexual orientation groups, the effect was present for female voices, too. The general pattern of findings suggests dispersion accuracy of speech stereotypes (i.e., the belief that members of one group do not differ regarding their speech characteristics) when judging speakers who represented the most extreme or the most typical examples of a given sexual orientation group. Hence, the evidence allows for two interpretations: First, listeners who judged sexual orientation used a consistent set of acoustic cues typical for the most typical speakers of a given sexual orientation group, and second, there are systematic acoustic differences between sexual orientation groups that are maximally different from each other.

Group stereotypes have been regarded as exaggerations of existing differences between social groups (e.g., [45]). The most important interaction in terms of explained variance was that self-ratings and other-ratings converged on very similar findings regarding bisexual or straight targets, whereas lesbian/gay voice averages based on other-ratings were perceived as more lesbian/gay than those based on self-ratings. Thus, people hold stereotypes of lesbian and gay speech more than of bisexual or straight speech. Moreover, the set of acoustic parameters that previous listeners based their judgments on, led to a more stereotypical perception in the present listeners: Voice averages of speakers who most clearly self-identified as lesbian/gay were judged as rather straight, whereas voice averages that consisted of speakers who were previously assessed by an independent listener group as most lesbian/gay were judged as most lesbian/gay. So stereotypical voices carry more lesbian/gay information than true voices of lesbians/gay men. Hence, this finding confirms our expectation that perceived sexual orientation differences were more pronounced in voice averages of speakers whose sexual orientation had been previously rated by others. In turn, this partially confirms Allport’s [45] old hypothesis that (speech) stereotypes are exaggerations with regard to dispersion.

By which means has the sexual orientation information been transmitted? The listeners just heard voices. Hence, the sexual orientation information has to be encoded within the speech signal of the voice averages. Members of one sexual orientation group seem to share a certain set of acoustic parameters that listeners use to correctly judge their sexual orientation. Descriptive findings support lay gender inversion theories by trend [27] because straight voice averages showed more gender conforming acoustic parameters. This can be partially attributed to voice averages based on stereotypically judged speech recordings only. Parameter-specific morphing could be used to systematically investigate this question which parameters the most informative ones about vocal sexual orientation. For instance, the relative importance of f0, formants, and timing on the perception of sexual orientation could be investigated using voice morphs between gay/lesbian and straight speakers varying only the parameter of interest from gay/lesbian to straight while keeping the residual at an uninformative intermediate morph level (cf. Skuk et al. 2014 for voice gender perception).

Our findings support previous research indicating the use of stereotypical information when judging others’ sexual orientation (see [48]). When assuring high stereotypical information of voice averages (i.e., selecting single voices for averages based on sexual orientation ratings by others), differences between sexual orientation groups were more pronounced than when less stereotypical information was present (i.e., selection based on speakers’ self-ratings). Moreover, differing mean f0 patterns provided further evidence: Mean f0 of voice averages with sexual orientation rated by others showed an overall stereotypical pattern whereas voice averages with self-rated sexual orientation did not.

Previous studies applying voice morphing techniques with regard to gender used simple stimuli, such as vowel-consonant-vowel dyads. In the present study, more complex sentence stimuli were morphed for the first time. Thus, our findings reflect acoustic and perceptual correlates of sexual orientation based on stimuli with increased ecological validity. Hence, we recommend the use of sentence stimuli in future voice morphing studies.

By using a voice morphing approach, we introduced a technique new to the sociophonetic research of sexual orientation. In order to test whether perceived sexual orientation differences between voice averages exist at all, we systematically selected voices for averaging. We tried to maximize sexual orientation information and to create maximally different voice averages by choosing those speakers for voice averaging who showed extreme scores on self-rated and other-rated sexual orientation (e.g., straight women who self-identified as exclusively straight only). By doing so, we increased the chance for finding support for dispersion accuracy at the outset. Hence, our findings do not imply that the sexual orientation of less extreme groups nor individuals can be judged accurately. Instead, our study should be taken as a starting point for future research that could test whether less typical sexual orientation information is still preserved after voice averaging, that is, when randomly selecting lesbians and gay men.

As another possible limitation of the present study, speakers were not blind to the purpose of the study. We can therefore not rule out some degree of self-selection among the speakers. Specifically, an increased number of lesbian/gay speakers who felt comfortable with their sexual orientation may have contributed to more stereotypical speaking patterns overall. Note however, that the issue of representativeness would not be resolved even if we had included all speakers from the initial sample to create the voice averages. Creating representative samples of speakers with respect to sexual orientation is an unrealistic undertaking [55–57]. Furthermore, the study’s announcement could have been appealing for listeners who believe that voices contain a lot of information about someone’s sexual orientation. Possibly these listeners wanted to show that a correct detection of sexual orientation based on voice is possible. However, neither subjective beliefs about the informative richness of cues to sexual orientation (see [58] for the visual domain) nor attitudes towards lesbians and gay men [59] are related to more correct responses. Moreover, it has to be mentioned that the present study was carried out in a particular culture, time, and language, and voluntary participants provided the speech samples that we used as the basis of our voice averages. Manners of speaking may vary with the language used, with role models, and with the sample investigated. Thus, the generalizability of our study’s findings needs to be tested by future research that may rely on the technological advances that we used here for the first time.

## Conclusion

The present study set out to answer the question whether there is a common set of acoustic parameters of sexual orientation in maximally different groups, or whether different individuals use different means to express their sexual orientation. To the best of our knowledge, this study was the first to use voice averages of complex stimuli (i.e., sentence-length utterances) to test this. We found an effect of sexual orientation across conditions, in line with the idea that the selected groups of most typical speakers used similar ways of expressing sexual orientation. Our findings do not allow conclusions regarding single speakers or less extreme groups. Additionally, the observed effect of sexual orientation was larger when voice averages were based on perceivers’ ratings than on self-ratings, demonstrating that stereotypes of gay or lesbian manners of speaking are exaggerations of the differences that truly exist between speakers.

## Supporting information

S1 File Wav: Bisexual female–sexual orientation rated by others. (WAV)

S2 File Wav: Bisexual female–self-rated sexual orientation. (WAV)

S3 File Wav lesbian female–sexual orientation rated by others. (WAV)

S4 File Wav: Lesbian female–self-rated sexual orientation. (WAV)

S5 File Wav: Straight female–sexual orientation rated by others. (WAV)

S6 File Wav: Straight female–self-rated sexual orientation. (WAV)

S7 File Wav: Bisexual male–sexual orientation rated by others. (WAV)

S8 File Wav: Bisexual male–self-rated sexual orientation. (WAV)

S9 File Wav: Gay male–sexual orientation rated by others. (WAV)

S10 File Wav gay male–self-rated sexual orientation. (WAV)

S11 File Wav straight male–sexual orientation rated by others. (WAV)

S12 File Wav straight male–self-rated sexual orientation. (WAV)
